# Supplementary figures and images for: Monocarboxylate transporter dependent mechanism is involved in proliferation, migration, and invasion of human glioblastoma cell lines via activation of PI3K/Akt signaling pathway
Source: PLoS One. 2024 Oct 30;19(10):e0312939. doi: 10.1371/journal.pone.0312939 (PMC11524508; doi:10.1371/journal.pone.0312939)

**Figure 1A (MCT4) U87**

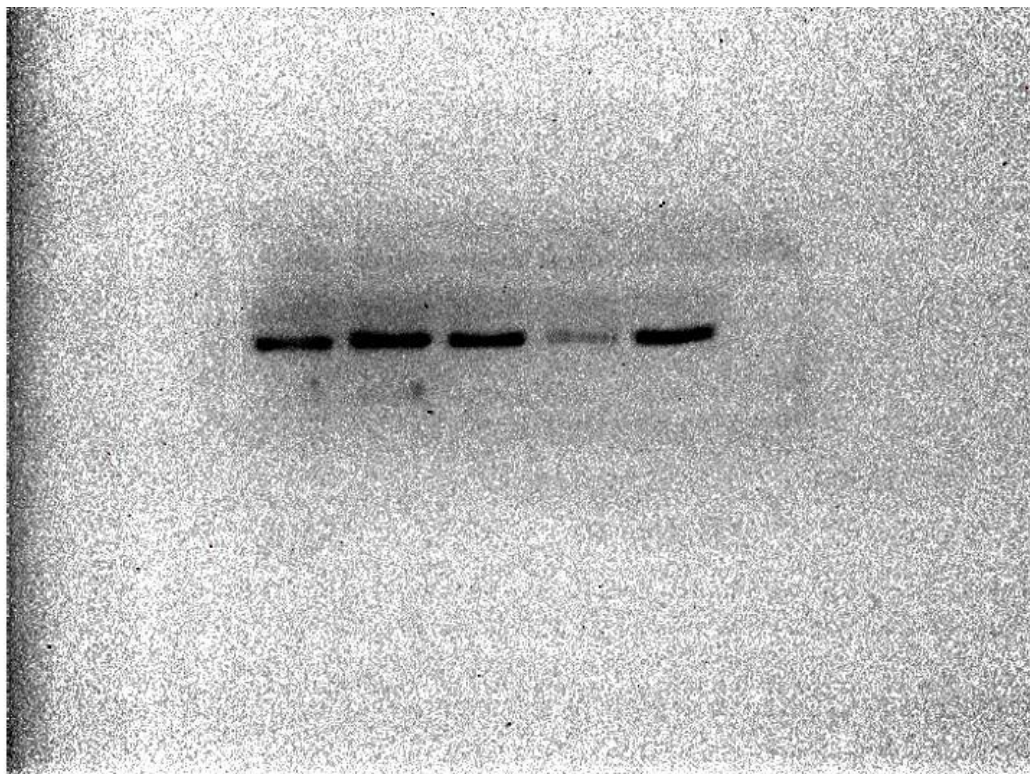

**Figure 1A (MCT4) U251**

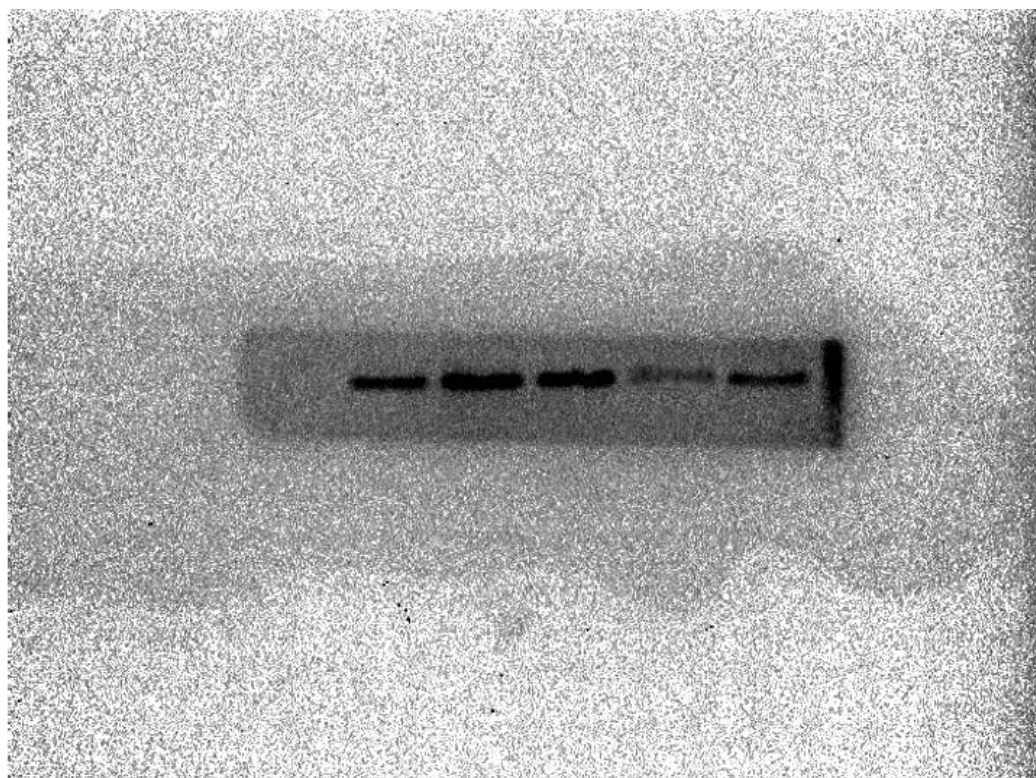

**Figure 1A tubulin**

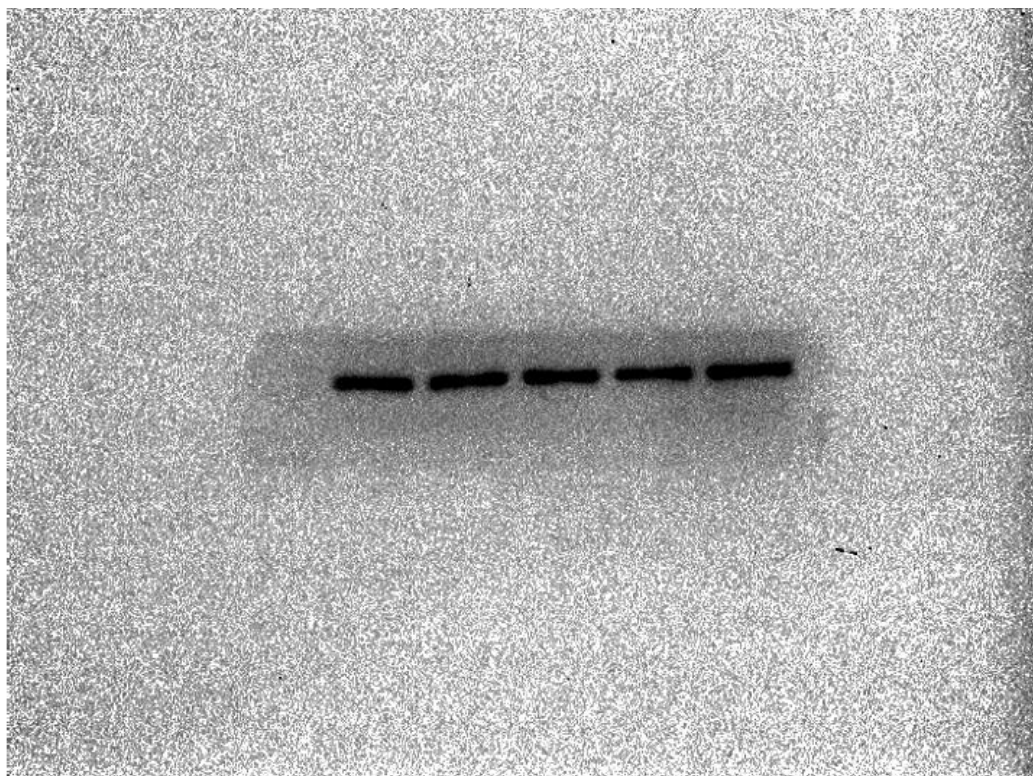

Supplement: S1 Raw images — Westernblot showing the entire gel for Fig 1A (MCT4 U87 and U251, α-tubulin), Fig 2A (MCT4 U87 and U251, CD147 U87 and U251, α-tubulin), Fig 4A (Phospho-Akt U87 and U251, Total-Akt U87 and U251, α-tubulin) were adapted from this western blot. (ZIP) [file pone.0312939.s001.zip › Supporting Information files/Raw image Fig1 A.pdf]

**Figure 2A-MCT4 (U87)**

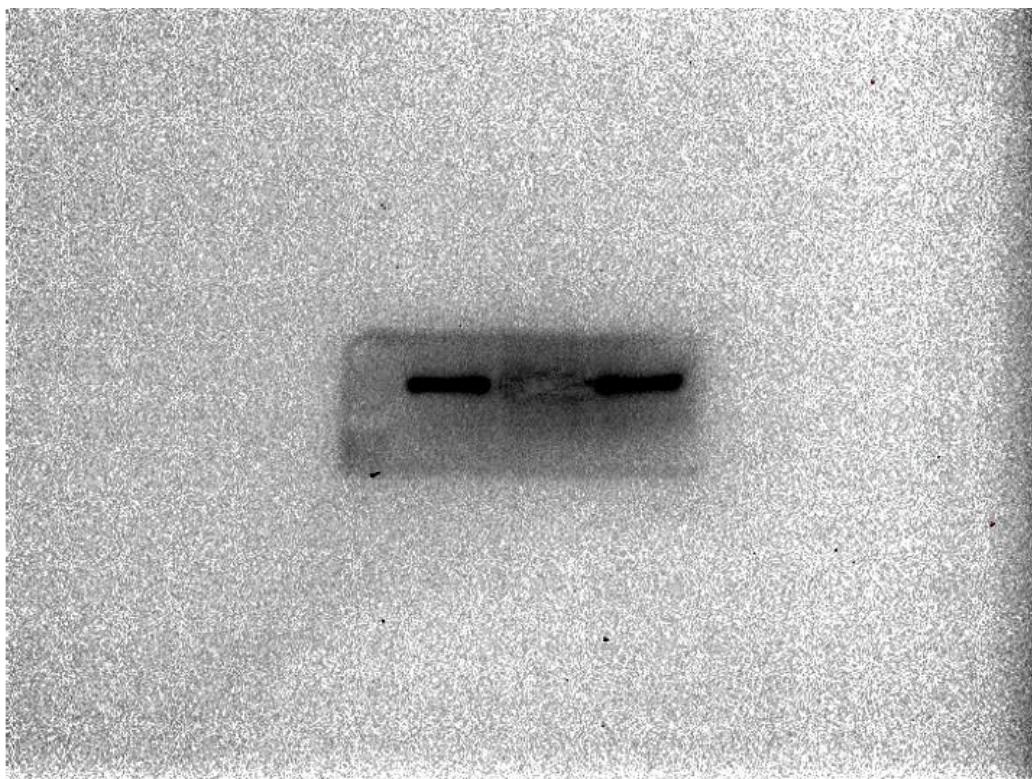

**Figure 2A-CD147 (U87)**

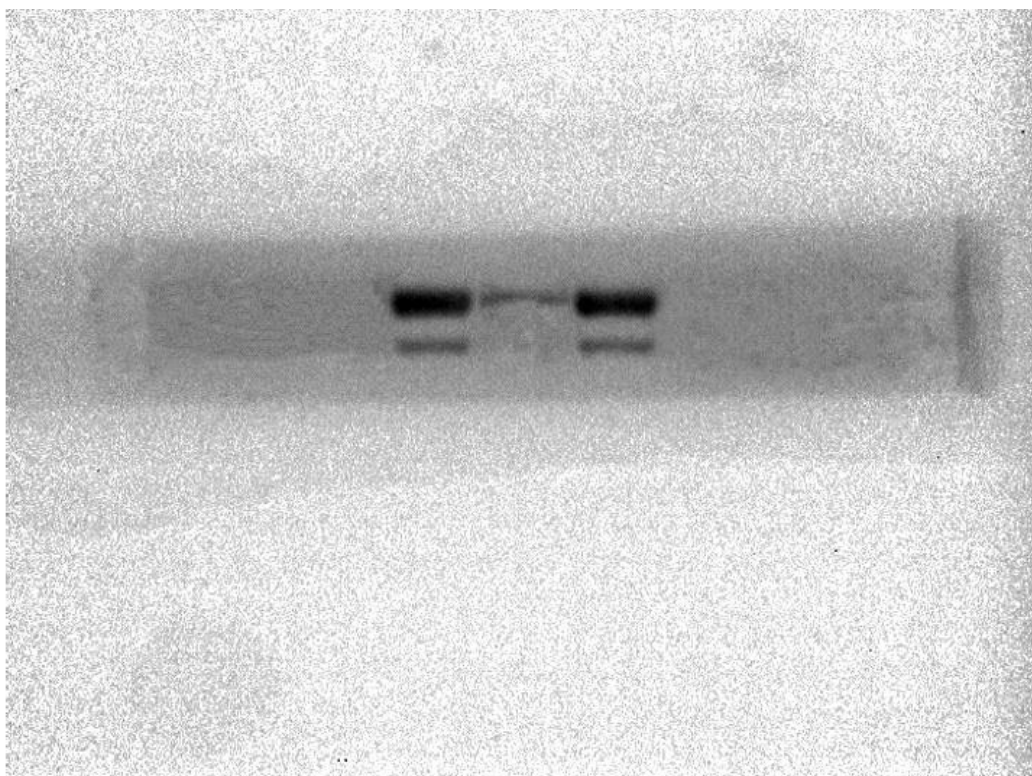

**Figure 2A-tubulin (U87)**

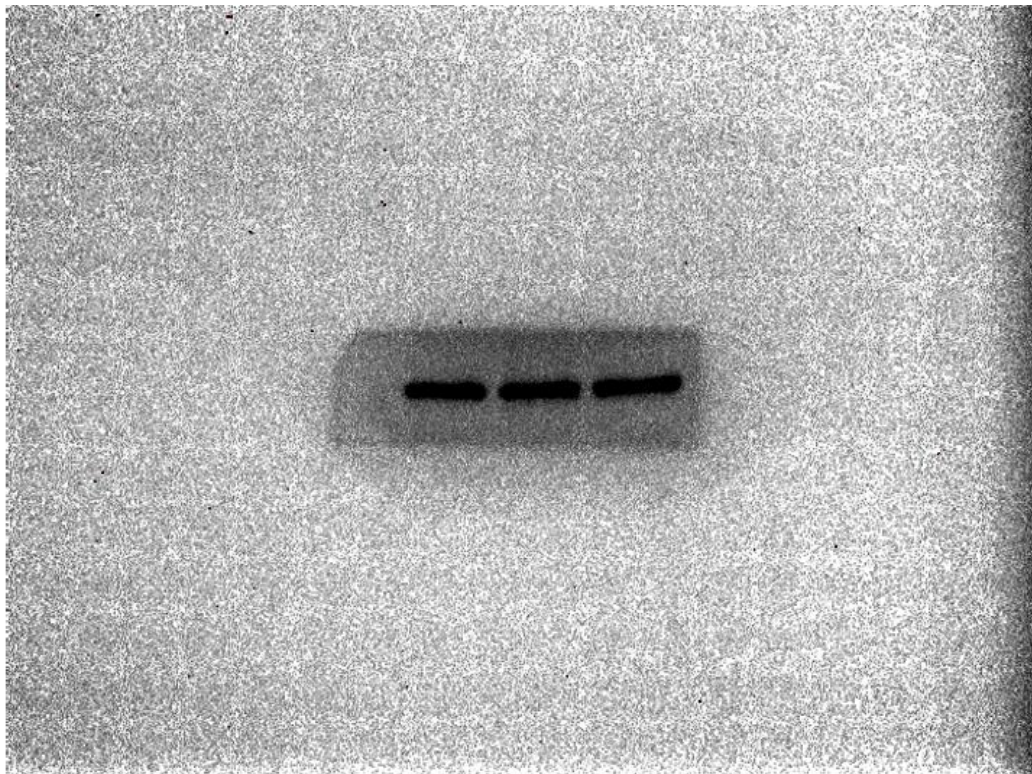

**Figure 2A-MCT4(U251)**

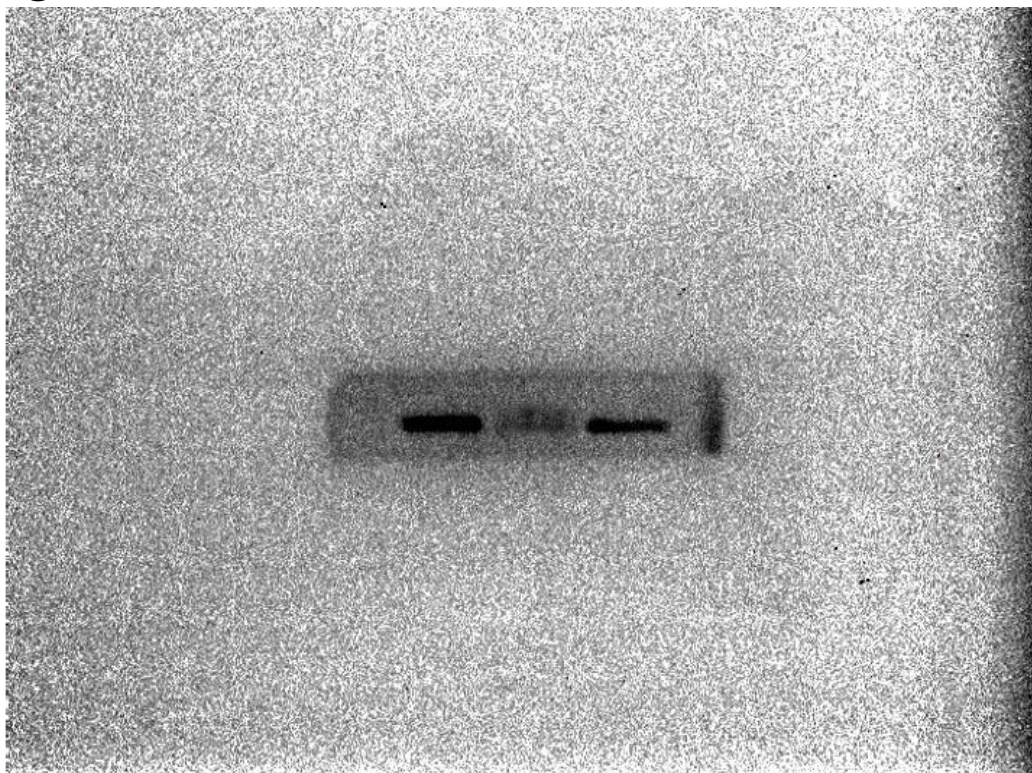

**Figure 2A-CD147 (U251)**

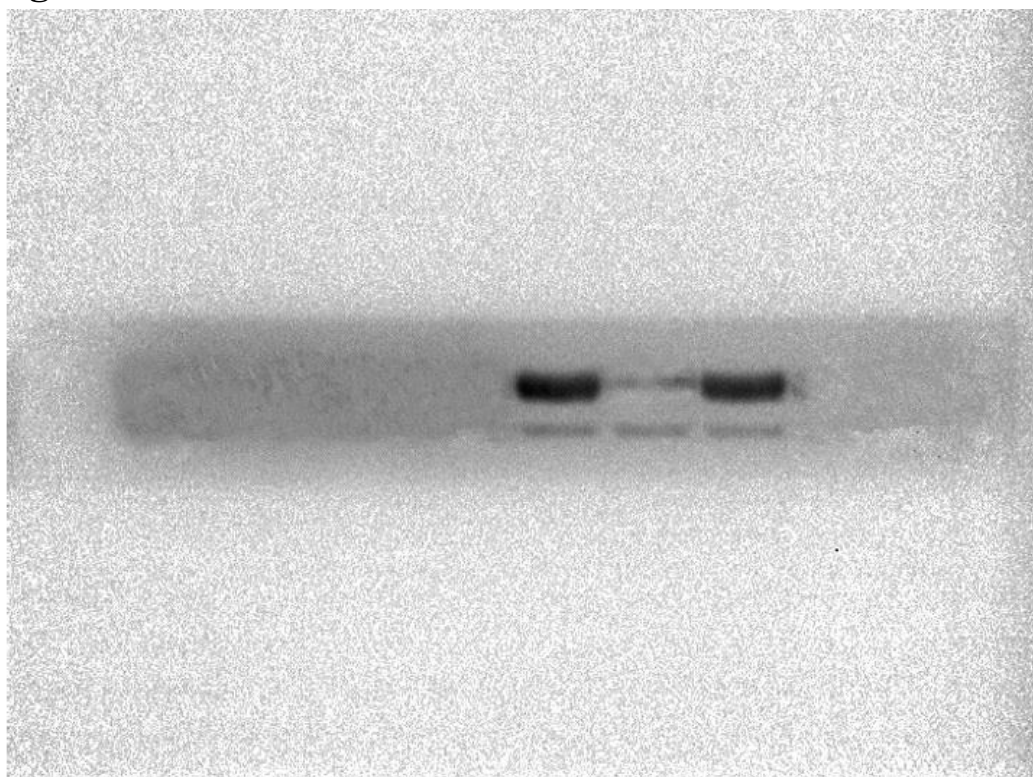

**Figure 2A-tubulin (U251)**

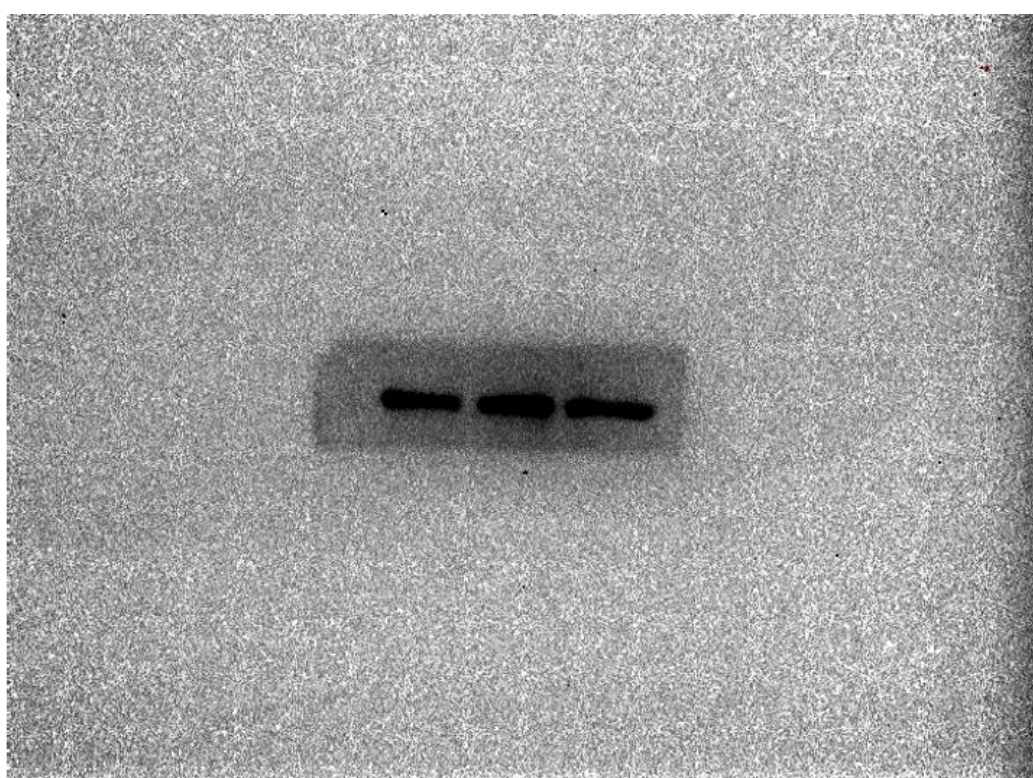

Supplement: S1 Raw images — Westernblot showing the entire gel for Fig 1A (MCT4 U87 and U251, α-tubulin), Fig 2A (MCT4 U87 and U251, CD147 U87 and U251, α-tubulin), Fig 4A (Phospho-Akt U87 and U251, Total-Akt U87 and U251, α-tubulin) were adapted from this western blot. (ZIP) [file pone.0312939.s001.zip › Supporting Information files/Raw image Fig2 A.pdf]
