# Supplementary material for: Monocarboxylate transporter dependent mechanism is involved in proliferation, migration, and invasion of human glioblastoma cell lines via activation of PI3K/Akt signaling pathway
Source: PLoS One. 2024 Oct 30;19(10):e0312939. doi: 10.1371/journal.pone.0312939 (PMC11524508; doi:10.1371/journal.pone.0312939)
Supplement: Attachment — Submitted filename: Response to the academic editor and reviewer(s).doc [file pone.0312939.s002.doc]

Response to the academic editor and reviewer(s)

I am very grateful to the editors and reviewers for their valuable comments. According to these comments, modifications were made in my manuscript.

**1. Response to reviewers.**

(1) Reviewer 1 pointed out that the statement in the discussion in lines 344-351 can hardly be supported by the manuscript in its present form. As the author of the manuscript, I personally agree with this view. This section is the concluding part of this study. Because changes in molecules downstream of the PIK3/Akt signalling pathway were not further tested, the MCT4/CD147 transporter complex mediated the biological characteristics of glioma cells in an Akt-dependent maner is speculative, and further testing of VEGF and related downstream molecules is needed to provide more adequate support in further studies.

(2) According to the comments of reviewers 1 and 2, the scientific background of the introductory section has been enriched, the novelty of this study has been presented, and relevant important concepts have been referenced. The corresponding bibliography of manuscript references has been extensively revised.

(3) Following the comments of reviewer 1, the following explanation is given regarding the very low increase of MCT4 in the lentiviral group. Results both section 3.1 and Figure 1 clearly showed that the increase of MCT4 was obvious after lentivirus transduction of cell lines, which showed statistical difference compared to other groups. The results of Western blotting and immunofluorescence detection of protein level and mRNA detection of gene level can confirm each other.

(4) Reviewer 1 pointed out that further determination of lactate levels to corroborate the biological function of MCT4 is a very professional suggestion. To this end, extracellular lactate content was additionally measured, and the trend of change was generally consistent with the change in MCT4 expression. Relevant content has been added to the Materials and Methods and Discussion sections.

(5) Clarification of lines 48-50 of the manuscript as follows: MCT4 and Basigin/CD147 have been reported to stimulate vascular endothelial growth factor (VEGF) through the phosphatidylinositol 3-kinase/Akt (PI3K/Akt) signalling pathway [12], which has been shown to mediate glioblastoma invasion and migration. This formulation has been confirmed in relevant studies[Tang Y, Nakada MT, Rafferty P, Laraio J, McCabe FL, Millar H, et, al. Regulation of vascular endothelial growth factor expression by EMMPRIN via the PI3Kakt signaling pathway. Mol Cancer Res. 2006; 4:371-377.]

(6) Cytofluorimetry is a technique for analysing cell properties by measuring fluorescent signals in cells or tissues. It is mainly used to study the physiological and pathological states of cells, including cell proliferation, apoptosis, and drug response. BrdU/EdU assays are two commonly used methods in cell proliferation assays, they track cell proliferation by labelling newly synthesised DNA, and are the most accurate method for detecting cell proliferation. Cell proliferation was also detected by MTT and Tepan blue cell assay, which are similar methods for the initial assessment of cell proliferation capacity with simplicity. In this study, MTT assay was chosen for the initial detection of tumour cell line proliferation, and for subsequent in-depth studies, the reviewer-suggested method must be chosen to accurately determine at the genetic level.

(7) This is clearly stated in both the abstract and introduction sections of the manuscript, MCT4 and Basigin/CD147 have been reported to stimulate vascular endothelial growth factor (VEGF) through the phosphatidylinositol 3-kinase/Akt (PI3K/Akt) signalling pathway. Therefore, I strongly agree with the reviewer's comment to include the measurement of VEGF in further in-depth studies so that the findings can be more fully substantiated.

(8) Thanks for the constructive comments from reviewer 1 and 2. GSK-3β is a multifunctional serine/threonine kinase belonging to the glycogen synthase kinase subfamily, which is a negative regulator of glucose homeostasis and is involved in energy metabolism, inflammation, endoplasmic reticulum stress, mitochondrial dysfunction, and apoptotic pathways. GSK-3β is a key downstream protein of the PI3K/Akt signalling pathway, and Akt can phosphorylate Ser9 of GSK-3β and inhibit its enzymatic activity. So Akt plays an important role in energy metabolism. In addition, Nrf2 is a key factor in cellular regulation of oxidative stress, and is the most sensitive signal for scavenging excessive intracellular ROS to combat oxidative stress. It is involved in various cellular activities, including maintenance of redox balance, proliferation, metabolism and apoptosis. It has been shown that the PI3K/AKT signalling pathway can regulate the activity of Nrf2, and the phosphorylation of AKT can further help Nrf2 to achieve the nuclear-plasma shuttling, which can lead to the enhancement of endogenous antioxidant in cells. The present study initially investigated the effect of monocarboxylic acid transporter proteins on the Akt signalling pathway. Thus, the detection of molecules downstream of the Akt signalling pathway, whether GSK-3β or Nrf2, could provide even stronger support for the manuscript's conclusions.

(9) In the present study, the expression of MCTs was modulated by lentiviral transfection and small RNA interference, and the corresponding changes in the Akt signalling pathway were initially investigated without using inhibitors. It is advisable to use sensitive inhibitors for further investigation of the downstream signalling pathways of the Akt signalling pathway.

(10) Line 180: The phrase ‘no drug treatment’ means no lentiviral treatment, i.e. a normal cultured tumour cell line is used as a blank control. Because of ambiguity, it has been changed to ‘without lentiviral transduction’.

(11) Paragraph 2.10 of revised manuscript: Add a detailed description of the transwell invasion assay in the manuscript as blow: Gently but firmly remove Matrigel from the inside of the transwell insert by use of a cotton tipped applicator to ensure all Matrigel and remaining non-migratory cells are removed.

(12) Paragraph 3.4 of revised manuscript: Comparison of the migration distances of the groups of cells in the manuscript has been able to visually represent the magnitude of the migration capacity of the cells.

(13) Processed images are provided in the article for clearer image comparisons. However, all data were analysed by normalising the original images with Image J software. Raw images of the Western blotting assay have been provided in the supporting information files.

(14) Manuscripts have been fully checked and corrected for spelling errors. The language of the manuscript has been revised and corrected by International Science Editing Ltd.

(15) All gene expressions in the RT-PCT in the manuscript were revised to italics.

(16) The acronyms in the abstract have been replaced with the relative extended names+abbreviations.

(17) Reviewer 2 suggested the use of the 3D spheroid invasion assay to provide stronger supporting evidence for the study, which is a good comment. In further studies, we will adopt the above suggestions in order to further improve the quality of the study.

(18) Glycosylation has a significant impact on the physicochemical properties and biological functions of proteins, including protein stability, folding state, and interactions with other molecules.

(19) As suggested by reviewer 2, the glycosylation of CD147 is interpreted as follows: CD147 is a transmembrane glycoprotein with a molecular weight of approximately 43-66 kDa. Highly expressed in a variety of tumour cells and tissues. Functional studies have revealed that CD147 promotes tumour cell infiltration and metastasis by inducing peripheral fibroblasts and tumour cells to produce a variety of matrix metalloproteinases (MMPs), including MMP-1, MMP-2 and MMP-9, which degrade the extracellular matrix. Highly glycosylated modifications are one of the important features of CD147.

**2. Response to Journal Requirements?**

(1) The manuscript has been completely revised to ensure meets PLOS ONE's style requirements.

(2) Research funding and role of funder statement have been added to both the revised manuscript and the cover letter.

(3) Raw images of Western blotting assays have been uploaded to the submission system as supporting information files, and other datasets used and/or analyzed during the current study cannot be shared publicly due to restrictions on the disclosure of research data of military units by the Ethics Committee. Data are available from the official website (https://www.gsinfo.net.cn/index.php/Institution/ywbmptzx.html) for researchers who meet the criteria for access to confidential data.

This statement has been revised in the revised manuscript.

(4) All images in the manuscript have been uploaded to the Preflight Analysis and Conversion Engine (PACE) digital diagnostic tool and revised to ensure PLOS requirements.

(5) Any funding-related text already removed from the manuscript.
