# Supplementary material for: Monocarboxylate transporter dependent mechanism is involved in proliferation, migration, and invasion of human glioblastoma cell lines via activation of PI3K/Akt signaling pathway
Source: PLoS One. 2024 Oct 30;19(10):e0312939. doi: 10.1371/journal.pone.0312939 (PMC11524508; doi:10.1371/journal.pone.0312939)
Supplement: Attachment — Submitted filename: Response to the academic editor and reviewer(s).doc [file pone.0312939.s003.doc]

Response to the academic editor and reviewer(s)

I am very grateful to the editors and reviewers for their valuable comments. According to these comments, modifications were made in revised manuscript.

(1) All the acronyms, such as MCT, CD147, PBKVARt, VEGF and siRNA had been defined at their first mention in both the abstract and the introduction.

(2) Line 70, the word “lacate” has been amended to “lactate”.

(3) Throughout the text of the manuscript, the word tumour is standardised to the British expression, ‘tumor’, but in the references, the American expression, ‘tumour’, is still used in some of the literature (reference 20 and 22).

(4) Closely in context, a more detailed description of the Transwell invasion assay method is provided in the Materials and Methods section, specifically describing the use of Image J software for counting migrating and invaded cells.

(5) To ensure consistency, line 296 and 362, the word “knockdown” has been amended to “interference”, line 359, the word “knockdown” has been amended to “silencing”.

(6) The method of immunoblotting image analysis with Image J software is described in detail in the Materials and Methods section.

(7) Three updated references on MCT4/CD147 role in glioblastoma have been to the Introduction, Materials and Methods, and Discussion sections, respectively. (Fernandez GB, et al. Cells, 2022. doi: 10.3390/cells11213467; Longhitano L et al. Front Oncol. 2022. doi: 10.3389/fonc.2022.871798. eCollection 2022; Kubelt C et al. Int J Mol Sci. 2020. doi: 10.3390/ijms21176254.)Reference numbers have been revised accordingly.

(8) Numerical data that are normally distributed can be analyzed with parametric tests, that is, tests which are based on the parameters that define a normal distribution curve. The one-way analysis of variance (ANOVA) is employed to compare the means of three or more independent data sets that are normally distributed. The quantitative data within the group in this study conformed to normal distribution, so one-way ANOVA was used for statistical treatment. Depending on results of homogeneity of Variance Test, the final choice of either Tamhane or Bonferroni correction was made for the between-group comparison results. The above is explained in the statistical analysis section.

(9) In the final part of the discussion section, expressions such as “suggest” and “hypothesis” are used, making it more cautious to draw research conclusions.
